# Plasmonic colloidal Au nanoparticles in DMSO: a facile synthesis and characterisation

**DOI:** 10.1039/d2ra03605c

**Published:** 2022-08-03

**Authors:** Volodymyr Dzhagan, Olga Kapush, Svitlana Plokhovska, Anastasiya Buziashvili, Yaroslav Pirko, Oleg Yeshchenko, Volodymyr Yukhymchuk, Alla Yemets, Dietrich R. T. Zahn

**Affiliations:** V. Lashkaryov Institute of Semiconductors Physics, National Academy of Sciences of Ukraine Kyiv Ukraine dzhagan@isp.kiev.ua; Physics Department, Taras Shevchenko National University of Kyiv 01601 Kyiv Ukraine; Department of Cell Biology and Biotechnology, Institute of Food Biotechnology and Genomics, National Academy of Sciences of Ukraine 04123 Kyiv Ukraine; Department of Population Genetics, Institute of Food Biotechnology and Genomics, National Academy of Sciences of Ukraine Osypovskogo str., 2a Kyiv 04123 Ukraine; Semiconductor Physics, Chemnitz University of Technology 09107 Chemnitz Germany; Center for Materials, Architectures and Integration of Nanomembranes (MAIN), Chemnitz University of Technology 09107 Chemnitz Germany

## Abstract

We report a new pathway for the synthesis of plasmonic gold nanoparticles (Au NPs) in a bio-compatible medium. A modified room temperature approach based on the standard Turkevich synthesis, using sodium citrate as a reducing and stabilizing agent, results in a highly stable colloidal suspension of Au NPs in dimethyl sulfoxide (DMSO). The mean NP size of about 15 nm with a fairly low size distribution is revealed by scanning electron microscopy. The stability test through UV-vis absorption spectroscopy indicates no sign of aggregation for months. The Au NPs are also characterized by X-ray photoelectron, Raman scattering, and FTIR spectroscopies. The stabilisation mechanism of the Au NPs in DMSO is concluded to be similar to that of NPs synthesized in water. The Au NPs obtained in this work are applicable as SERS substrates, as proved by common analytes. In terms of bio-applications, they do not possess such side-effects as pronounced antibacterial activity, based on the tests performed on non-pathogenic Gram-positive or Gram-negative bacteria.

## Abstract 2

We report on the synthesis of stable plasmonic gold nanoparticles (Au NPs) in dimethyl sulfoxide (DMSO) and demonstrate that the AU NPs are biocompatible and function as SERS-active substrates.

## Graphical abstract

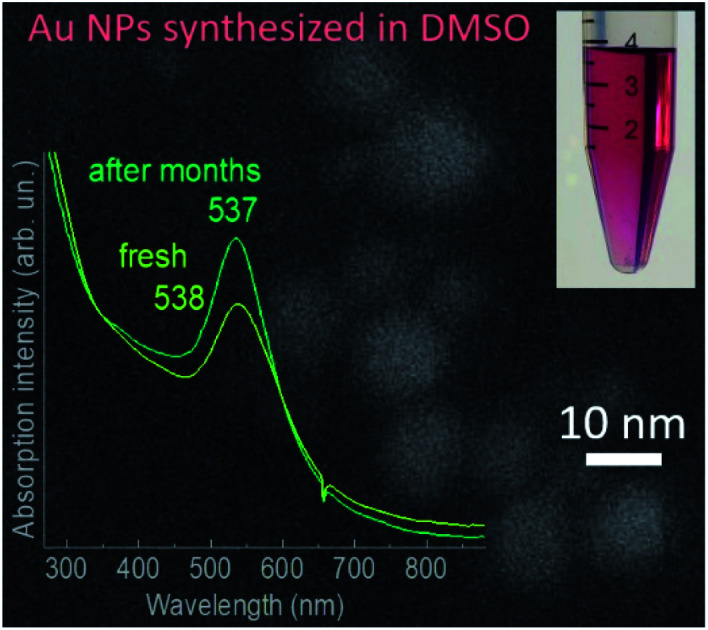

## Introduction

Noble metal nanoparticles show remarkable physical and chemical properties, which determine their extensive use in various fields of optics, electronics, biology, and nanomedicine. Especially valuable is their ability to support localized surface plasmonic resonances (LSPRs) – collective oscillations of free charge carriers, excited by the electric field of light.^1^ The applications of LSPR include surface-enhanced Raman scattering (SERS),^2–7^ plasmon-enhanced photoluminescence,^8–12^ and related optical sensing techniques,^9,13–19^ bio-imaging,^20^ photothermal approaches in anti-tumor therapy,^21,22^ plasmon-enhanced energy-harvesting,^23^*etc.* Numerous methods of synthesis of colloidal NPs have been developed so far,^1,2,14,24–27^ with the facile methods of producing bio-compatible NPs being one of the main research focuses in the community.^28–30^

Among the most often used syntheses is the one leading to a colloidal system of spherical gold NPs based on a simple reaction reducing auric acid with either sodium citrate or sodium borohydride.^31^ Sodium citrate is particularly interesting, as it is cheap and nontoxic and it does not only act as the reducing agent but its negatively charged carboxylates are also responsible for the charge stabilization of the AuNPs.^32^

In addition to imparting specific functionalities to nanoparticles, their dispersion in water or any other biocompatible solvent is crucial for their use in biomedical or biological applications. Therefore, water should be the primary solvent used to disperse functionalized nanoparticles. However, in many cases, it would be beneficial to have a range of other common solvents, dimethyl sulfoxide (DMSO) being one of them. DMSO is a broadly used solvent, applied in different areas from bio- and medical research^33,34^ to electronic and energy application,^35^ but it has been only rarely used as a medium for synthesis of colloidal Ag NPs,^36^ Au NPs,^37,38^ or semiconductor NPs.^39,40^ In ref. 37 a multi-stage synthesis of Au NPs was developed, using cetylpyridinium chloride, hexadecyltrimethyl-ammonium bromide, hydroquinone, ethylene glycol, and polyvinylpyrrolidone. The approach of ref. 38 included the synthesis of new phosphonium-based ligands, which behaved as cationic masked thiolate ligands in the functionalisation of Au NPs dispersible in water and DMSO. In ref. 41 ultrasound assisted synthesis of Au NPs in DMSO without any additional stabilizer was reported. It apparently produced only aggregates of highly polydisperse NPs, as may be concluded from their scanning electron microscopy images and the absence of optical images of the NP solution and UV-vis absorption spectra.

In this work, we obtained a highly stable “colloidal” suspension of Au NPs in dimethyl sulfoxide (DMSO). A modified room temperature approach based on the standard Turkevich synthesis (Turkevich *et al.* 1951), using sodium citrate as a reducing and stabilizing agent, results in stable NP colloids with a fairly low size distribution. The possible stabilisation mechanism of the Au NP in DMSO is discussed based on the results of X-ray photoemission spectroscopy (XPS) and Fourier-transform infrared (FTIR) measurements. In view of perspective applications, the Au NPs obtained were tested as SERS substrates. It was also evaluated how environmentally friendly the synthesized Au NPs are and whether they have any side effects on living organisms in the ecosystem, such as bacteria, which are most often found in nature or used as model objects in different studies. Further prospective advantages of the present NPs are seen in the applications where aqueous analogues cannot provide appropriate wetting of certain substrates, miscibility with organic solvents or dissolving organic molecules, or other materials not solvable in water, which is a necessary step of the sensing (including SERS) or forming composite materials (or conjugates).

## Materials and methods

### Synthesis of Au NPs

Hydrogen tetrachloroaurate(iii) trihydrate (HAuCl_4_·3H_2_O, 99.995% trace metals basis), trisodium citrate dihydrate (HOC(COONa)(CH_2_COONa)_2_ × 2H_2_O, ≥99.0%), dimethylsulfoxide (DMSO, melting temperature of −20 °C) were purchased from Sigma-Aldrich. All these chemicals were used as received. All glassware was cleaned with aqua regia (3 : 1 v/v HCl (37%) : HNO_3_ (65%) solutions) and then rinsed thoroughly with Milli-Q water before use. Caution: aqua regia solutions are dangerous and should be used with extreme care; never store these solutions in closed containers. Milli-Q water (18 MΩ cm, Millipore) was used to prepare all solutions in the experiments. The aqueous solution of HAuCl_4_ (1 wt%) was prepared and stored at *ca.* 4 °C before use.

Gold nanoparticles were synthesized by citrate reduction of hydrogen tetrachloroaurate(iii) trihydrate solution mixed with trisodium citrate dihydrate following the modified Turkevich method (Turkevich *et al.* 1951).^42^ In a typical Au NP synthesis, 0.1 mL of 1 M aqueous solution HAuCl_4_·3H_2_O was diluted with 10 mL of DMSO and heated until it begins to boil. To avoid contamination and evaporation of the solvent during the synthesis, a disposable Petri dish was used to cover the flask. After the HAuCl_4_ solution reached the boiling point under ambient pressure, a specific volume of HOC(COONa)(CH_2_COONa)_2_ × 2·H_2_O aqueous solution was rapidly injected into the HAuCl_4_ solution. The molar ratio (MR) of HOC(COONa)(CH_2_COONa)_2_ × 2·H_2_O to HAuCl_4_ was the primary factor controlled to achieve the desired particle size. The synthesis was complete when the color of the suspension no longer changed. Typically, the reaction took 2–5 min depending on the MR. The sample was cooled naturally to room temperature.

### Physical characterisation

Optical absorption spectra were recorded from NP solutions in 1 mm wide plastic cuvettes using a StellarNet Silver Nova 25 BWI6 Spectrometer. Scanning electron microscopy (SEM) was performed from NP samples dried from a colloidal solution on a Si substrate using a Tescan Mira 3 MLU. Dynamic light scattering (DLS) measurements were performed with a particle sizer and Zeta Potential Analyzer NanoBrook Omni (Brookhaven Instruments) equipped with a 532 nm laser. XPS measurements were performed with an ESCALAB 250Xi X-ray Photoelectron Spectrometer Microprobe (Thermo Scientific) equipped with a monochromatic Al Kα (*hν* = 1486.68 eV) X-ray source. Pass energy of 200 eV was used for survey spectra, 40 eV for Auger spectra, and 20 eV for high-resolution core-level spectra (providing a spectral resolution of 0.6 eV). Spectra deconvolution and quantification were performed using the Avantage Data System (Thermo Scientific). The linearity of the energy scale was calibrated by the positions of the Fermi edge at 0.00 ± 0.05 eV, Au 4f_7/2_ at 83.95 eV, Ag 3d_5/2_ at 368.20 eV, and Cu 2p_3/2_ at 932.60 eV measured on *in situ* cleaned metal surfaces. To prevent charging, the NP samples were measured using a built-in charge compensation system. Infrared absorption spectra were recorded from NPs dried on double-side polished Si using transmission geometry of a VERTEX 80v FTIR spectrometer (Bruker) equipped with a DLaTGS detector and a KBr beam splitter. Raman spectra were excited with 457, 532, or 671 nm single-longitudinal-mode solid-state lasers, with a power density on the samples of less than 10^3^ W cm^−2^, in order to preclude any thermal or photo-induced modification of the samples. Dispersion of the spectra was performed using a single-stage spectrometer (MDR-23, LOMO) with a spectral resolution of 6 cm^−1^ (as measured by the peak width of a single crystal Si substrate and the Rayleigh peak). Detection of the spectra was performed with TE-cooled (−60 °C) CCD detector (Andor iDus 420). At least 4 spots were probed on each sample studied, in order to ensure that the spectra are representative.

### Study of antibacterial activity

#### Bacterial strains and growth conditions

Three strains of Gram-positive bacteria (*Bacillus subtilis* IMB B-7445, *Bacillus thuringiensis* IFBG 800, *Corynebacterium glutamicum* IFBG B-216) and four strains of Gram-negative bacteria (*Agrobacterium tumefaciens* GV3101, *Escherichia coli* DB31, *Rhizobium leguminosarum* NRRL B-4403, *Bradyrhizobium japonicum* IMB B-7538) from the collection of microorganisms of the Institute of Food Biotechnology and Genomics, National Academy of Sciences of Ukraine, were used for antibacterial assays. The cultivation media for bacterial stains was solid/liquid Luria–Bertani (LB) bacteria growth media (Sambrook & Russell, 2001). Bacterial cultures were prepared by picking a colony from 24 h-old LB plates and placing them into a liquid LB medium. Cultures were grown overnight and continuously shaken at 100 rpm at 27 °C (*C. glutamicum*, *A. tumefaciens*, *R. leguminosarum*, *B. japonicum*) or at 37 °C (*E. coli*, *B. subtilis*, and *B. thuringiensis*). The overnight cultures of each bacteria strain were diluted with LB medium to an optical density OD_600_ = 0.1 and used for disk diffusion assay.

#### Antibacterial effect of Au NPs

The antibacterial test was carried out using the disk diffusion method (Klančnik *et al.* 2010;^43^ Buziashvili *et al.*, 2020^44^) with some modifications. For this assay, 1 mL of each bacterial suspension (OD_600_ = 0.1) was uniformly spread on the surface of a solid LB medium in a Petri dish (9 cm in diameter). Four sterile disks of filter paper (*d* = 5 mm, Whatman®, Sigma-Aldrich, USA) were placed on the surface of each plate with bacteria. Stock solution (SS) of synthesized Au NPs was used to prepare several dilutions (1 : 0, 1 : 1, 1 : 2) using sterile distilled water. Each disk was loaded with 10 μL of samples: different dilutions/concentrations of Au NPs (1:2SS, 1:1SS, 1SS or 0.25SS, 0.5SS, 1.0SS or 73.5, 147 and 294 μM, or 14.45, 28.5 and 57 μg mL^−1^), DMSO (as positive control) and sterile distilled H_2_O (as negative control). Petri dishes with bacteria and loaded disks were incubated for 16 h under appropriate cultivation conditions. The antibacterial effect was evaluated by measuring the diameter of growth inhibition zones using ImageJ software (https://imagej.nih.gov/ij/). At least three replicas of each Au NPs concentration and positive/negative controls were performed.

#### Statistical analysis

The reliability of the results was confirmed with the one-way analysis of variance (ANOVA) test. Significant differences among means were considered at *P*-value of <0.05 level. Statistical data processing was performed with the use of Microsoft Office Excel 2010 software.

## Results and discussion

**Fig. 1:**
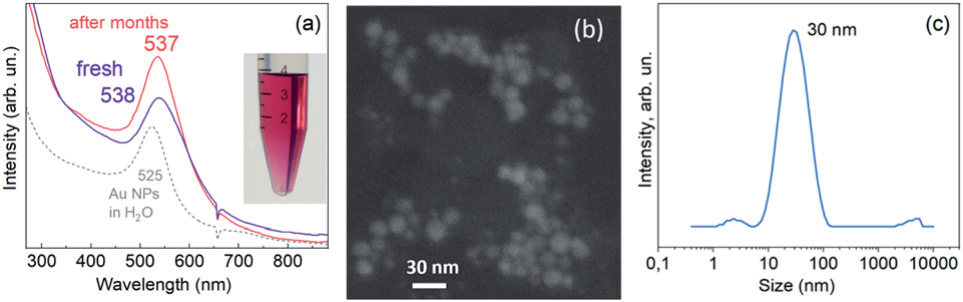
UV-vis absorption spectrum of Au NPs in DMSO (a), SEM image (b), and DLS size distribution (c). The inset in (a) shows the NP solution after several months of storage. The dotted curve shows the spectrum of Au NPs synthesized by a standard Turkevich method in water.

As-synthesised Au NPs were first characterised with UV-vis absorption spectroscopy because it is the most convenient and informative tool to prove the formation of the particles with LSPR.^1^ The observed distinct absorption band at about 538 nm (Fig. 1a) is characteristic of the LSPR absorption in Au NPs,^1^ however, it does not provide a precise estimate of the size of NPs formed. Therefore, a direct method of size determination was applied, namely scanning electron microscopy (SEM). A representative SEM image of the samples is shown and proves the formation of nearly spherical NPs with a moderate size dispersion and an average diameter of 15 ± 5 nm (Fig. 1b).

Besides the size of the inorganic (metallic) part of the colloidal NPs, it is important to have information about the molecular layer on their surface, which provides the colloidal stability of the NPs and ligands and determines their functionality. The method of dynamic light scattering (DLS) delivers the hydrodynamic size of the NPs, which includes the shell of the ligand bound to the NP surface. The obtained mean hydrodynamic diameter of our Au NPs, about 30 nm (Fig. 1c), is notably larger than the size of the inorganic (metallic) part determined with SEM. Such a large discrepancy can hardly be explained by the ligand shell alone but it is most likely due to the formation of aggregates of NPs of the smallest size.

The colloidal stability of the obtained NPs was assessed by storing the suspensions in preset temperature and humidity conditions for five months. The solution of Au in DMSO, similarly to Au NPs in water, is a typical electrostatically stabilized colloid so that the electrostatic component is determinant in securing the stability of the colloid towards aggregation and precipitation. In the literature, Au NPs synthesized following the Turkevich method are referred to as “citrate-stabilized”. We adhere to this terminology, although we would like to note that this terminology is valid as long as the electrostatic stabilization is taken into account. Because the citrate anion acts as both a reducing agent and stabilizer, its concentration is a critical parameter that affects the reduction rate of the ions and the growth rate of the NPs. With the citrate being used as a reducing agent, the formation of Au NPs is expected to occur by the following reaction:

1 2HAuCl_4_ + 3Na_3_C_6_H_5_O_7_ → 2Au + 3Na_2_C_5_H_4_O_5_ + 3CO_2_ + 3NaCl + 5HCl

In addition, as a result of the reduction reaction in solution oxidation products may be formed such as Na_2_C_5_H_2_O_4_ or Na_2_C_5_H_2_O_4_. However, it should be noted that all these reaction products, except carbon dioxide, can act as a reducing agent of chloroauric acid, so it is difficult to determine at what stage the process stops, and what is the ratio of these components in the final solution.

Citrate is an important stabilizing, reducing, and complexing agent in the wet chemical synthesis of noble metal nanoparticles and other metals.^36,45^ Despite a particularly large number of investigations focused on Cit-Au NPs, the structural details of citrate anions adsorbed on the Au NP surface are still being investigated and discussed.^45–47^ Au-citrate coordination in the liquid is supposed to occur mostly bidentate and is simply controlled by its protonation state.^32^ In ref. 45 it was found that, contrary to the common view, the species adsorbed on Ag NPs are, in large part, products of citrate decomposition comprising an alcohol group and one or two carboxylates bound to the surface Ag atoms, and minor unbound carboxylate group. These products may also be mixtures of citrate with lower molecular weight anions. No ketone groups were specified, and very minor surface Ag(i) and Fe (mainly, ferric oxyhydroxides) species were detected. Moreover, the content of adsorbates varied with Ag NPs size and shape. Several forms of citrate bonded to the Ag surface through carboxylate groups were proposed mainly on the base of surface-enhanced Raman scattering (SERS) and DFT simulation.^45^ A very informative method regarding the NP-ligand systems is X-ray photoelectron spectroscopy (XPS).^45–49^

The challenge with obtaining XPS data on colloidal Au NPs is twofold. First, as any colloidal NP sample deposited and dried on the substrate for the measurement, it may be undergoing charging during the measurement. The reason is poor electrical contact between the individual NPs and the substrate, resulting in an uncompensated positive charge in the NPs due to the loss of photoelectrons. Secondly, the most convenient substrate for the XPS measurements of colloidal samples is a thin gold film on top of a Si wafer or any other conductive enough material. Gold is preferred because it is chemically inert. Therefore, it does not get oxidised noticeably and does not react with deposited molecules (except sulphur). Moreover, it does not have its oxide layer and thus does not hinder the analysis of oxygen species in the sample. For measurement of Au NPs, however, the contribution of the substrate overlaps with that of the NPs themselves, requiring additional measurement, with other substrates. Therefore, in our study, we performed XPS measurements of Au NPs on Au and Si/SiO_2_ substrates. The former is more reliable for avoiding the charging effects, while the latter allows the quantification of Au content in the sample. Although the oxygen from SiO_2_ can interfere with the citrate oxygen peaks, this problem can be easily circumvented simply by picking up for analysis only spectra without Si contributions (meaning that there is no contribution of the SiO_2_ substrate to the O 1s range as well).

**Fig. 2:**
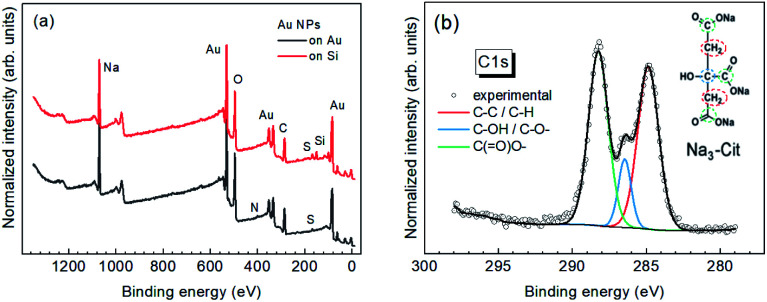
(a) Survey XPS spectra of the Au NPs on Au and Si substrates (normalized to Au peak intensity). (b) High-resolution XPS spectrum of carbon (C 1s), with deconvolution into components. The inset shows the schematic of the Na-citrate molecule and the expected contribution of bonds to the XPS C 1s spectrum.

The survey XPS spectra of the Au NPs on Au and Si substrates reveal only the elements expected for the samples (Fig. 2a): Au, C, O, Na (from sodium citrate), Si (from Si substrate), S (DMSO residuals), and N (minor surface contamination from the air). The C : O : Na ratio obtained from the survey XPS spectra is 2.5 : 2.5 : 1 on Au substrate and 2.6 : 3:1 on Si. The obtained ratio on Si is in perfect agreement with the C : O : Na ratio expected for Na-citrate based on its formula C_6_H_5_O_7_·3Na – 2.6 : 3 : 1. Apparently, Na-citrate can be considered as the main stabilizer of the Au NPs synthesized in DMSO. Further details on the molecular species and the mechanism of stabilization of Au NPs can be gained from the high-resolution XPS spectra. The spectrum of carbon (Fig. 2b), reveals three components that can all be attributed to the carbon atoms in the Na-citrate molecule (inset). The ratio of the components, C_285_ : C_286_ : C_288_ = 4 : 1 : 4, deviates, however, from the one expected for this molecule, 2 : 1 : 3, indicating that a minor part of the stabilizer molecules in our samples are products of citrate decomposition, in agreement with previous results on Au NPs stabilized by citrate in water.^45^

**Fig. 3:**
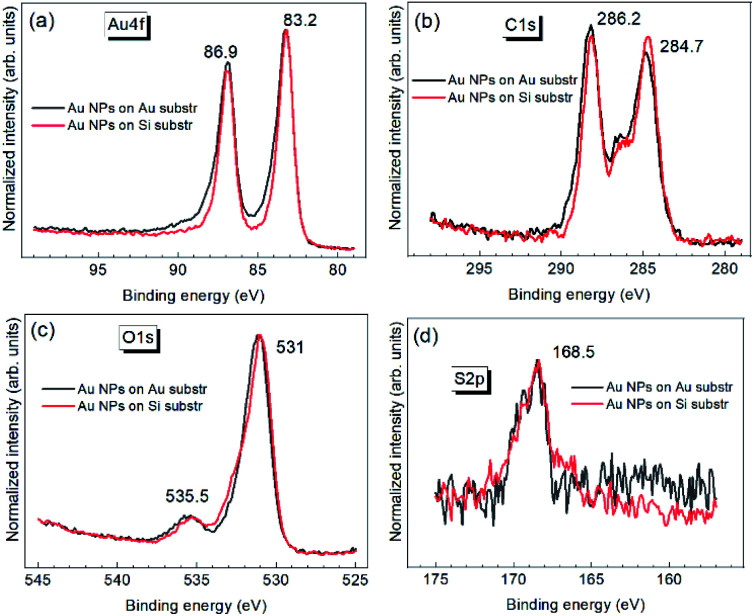
High-resolution XPS spectra of Au NPs on Au and Si substrates: Au 4f (a), C 1s (b), O 1s (c), S 2p (d).

The commonly characterized core-level spectrum of Au is the 4f doublet (Fig. 3a), with its 4f_7/2_ component BE at 84 eV for bulk samples.^50,51^

For Au NPs and other types of nanostructures, this characteristic XPS feature was reported both at much higher and much lower BEs. The higher binding energies are related to (contribution of) oxidized Au species on the surface, Au^+^, and Au^3+^.^49^ The lower BEs are usually explained by charge transfer from the ligands, substrate, or other environments.^52^ Alternatively, the negative core level shift for the surface atoms of bulk Au is explained by an initial state effect due to 6s → 5d charge reorganization for less coordinated surface atoms (bulk configuration 5d^9.6^6s^1.4^).^51^ The Au 4f_7/2_ peak position of 83.2 eV observed for Cit-Au–DMSO NPs (Fig. 3a) indicates that the latter mechanism can be involved. Nevertheless, the charge transfer with the stabilizer molecules cannot be excluded at the moment.

The XPS spectra measured on Au and Si coincide well for all four elements (Fig. 3a–d), indicating that the continuous area of the NP film was probed, without a noticeable contribution of the substrate, and, on the other hand, the film was thin enough to avoid charging.

**Fig. 4:**
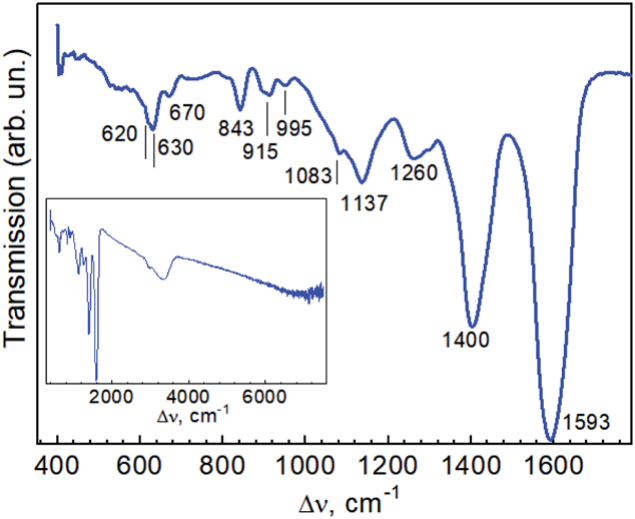
Infrared transmission spectrum of Au NPs.

IR absorption spectra of two Au-cit–DMSO NP samples are shown in Fig. 4. Typically, the carboxylate group exhibits an asymmetric and symmetric stretching vibrations around 1500–1630 and 1305–1415 cm^−1^, respectively.^46,53^ The contribution of a broad band at 1635 cm^−1^ due to water-bending vibration could be identified in the spectral range of interest by its disappearance during the drying of the sample.^46^ The IR absorption spectra (acquired in ATR geometry) of purified Cit-AuNPs studied in ref. 46 showed three distinct peaks, the asymmetric COO^−^ stretching vibrations, *ν*_asy_(COO^−^), at 1611, 1593, and 1558 cm^−1^, and three other peaks assigned to symmetric COO^−^ stretching vibrations, *ν*_sym_(COO^−^), at 1405, 1394, and 1370 cm^−1^. From comparison with the spectrum of pure trisodium citrate (Na_3_Cit) possessing the *ν*(COO^−^) at 1575 and 1385 cm^−1^ the authors of ref. 46 concluded three different configurations of the adsorbed citrate molecule on the Au NPs. The band at 1400 cm^−1^, the same we observe for Cit-Au–DMSO NPs (Fig. 4), was established in ref. 46 to be related to free citrate molecules, in particular for those with intramolecular hydrogen bonding (of their carboxylate group) with the hydroxyl group (of another citrate molecule). Therefore, we may assume a multilayer aggregation of the citrate molecules around the Au NPs, which could partially explain also the deviation between the NP size derived from SEM and DLS. However, a peak around 1700 cm^−1^, which is a spectral indication of a hydrogen bonding between free molecules and those bound to the NP surface,^46^ is not observed in our case. It is not straightforward to get experimental evidence of the difference between the NP size provided by DLS and SEM. The shell of stabilizer molecules, which exists in the solution and contribute to the hydrodynamic size, measured by DLS, may be partially destroyed upon drying the solvent (DMSO), *e.g.* for SEM measurements. Our analysis of the literature shows that even for one and the same sort of stabilizer, sodium citrate, there are many works where the NP size determined from DLS (*d*_DLS_) is close to that determined by SEM or TEM) (*d*_EM_).^54–56^ However there are also works, where *d*_DLS_ is much larger than *d*_EM_^57–59^ or even reveal the unexpected situation with *d*_DLS_ < *d*_EM_.^60–62^ Therefore, this issue is apparently an object of a separate study that is beyond the scope of the present paper.

The spectrum of “bulk” citrate recorded in ref. 63 exhibited bands at 1079, 1157, 1194, 1269, 1279, 1399, and 1591 cm^−1^. For citrate bound to Au NPs in ref. 63 the latter two features shifted to 1382 and 1638 cm^−1^ and notably weakened in intensity, while stronger peaks at 1261 cm^−1^ (sharp), 1098 cm^−1^ (broad), and 804 cm^−1^ (sharp) appeared. A rough estimation performed in ref. 63 for 24 nm Au NP gave 3000 citrate molecules per NP after purification, which corresponds to only half surface coverage. For the (not purified) Au NP samples, which contained an excess of citrate, an intermediate situation was observed – the latter three peaks (1261, 1098, and 804 cm^−1^) were present along with peaks at 1399 and 1591 cm^−1^.^63^ This spectral pattern is closer to that observed in our work for NPs in DMSO (Fig. 4), compared to other works,^46,53^ if we assume the correspondence between the peaks at 840 cm^−1^, 1137 cm^−1^, and 1260 cm^−1^ in our work with 804 cm^−1^, 1098 cm^−1^, and 1261 cm^−1^ observed in ref. 63. The latter differences in the spectra of Au NP synthesized in DMSO compared to those reported in the literature for citrate-stabilized Au NPs in water can be caused by the effect of the solvent on the stabilization of the NPs by citrate molecules. In ref. 46 it was shown that the pH of the solution can have a significant effect on the citrate adsorption geometry on the Au NP surface. From the detection of sulphur in the XPS spectra, we can conclude that a small quantity of DMSO molecules remains in the sample after drying the solution on the substrate. At a closer look at the 1137 cm^−1^ peak in our spectra, it contains shoulders at 1083 and 1112 cm^−1^ (Fig. 4). Therefore, the three vibrations in this range can be due to the contribution of free and differently bound citrate molecules.

**Fig. 5:**
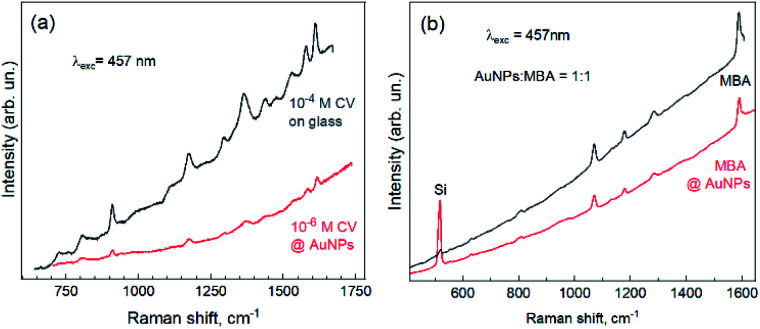
An illustration of the plasmon-enhanced Raman spectra (SERS) of crystal violet (a) and mercapto benzoic acid (b) by Au NPs synthesized in this work.

As one of the main applications of metal NPs is surface-enhanced Raman scattering of low quantities of organic molecules,^1,3,64^ we performed a preliminary test of the new synthesized NPs as a SERS substrate for detection of a low concentration of popular analytes. In particular, we observed enhancement for crystal violet (Fig. 5a) and mercapto benzoic acid (Fig. 5b) molecules mixed in a solution with colloidal Au NPs and dried on the Si substrate.

Note that the fact of enhancement in the case of MBA can be inferred based on an order-of-magnitude stronger intensity of the substrate (Si) peak, indicating that much smaller molecular coverage in the case of MBA with Au NPs gives a comparable signal intensity to the thick pure MBA film.

Since the synthesized Au NPs can be used for further development of a SERS-nanoplatform for the detection of different bacterial species and microbial contaminants, as well as in other bio-related applications, it is important to establish the presence or absence of possible side effects, namely the toxic effect on certain bacteria. Therefore, an antibacterial activity of Au NPs against seven different Gram-positive (*B. subtilis*, *B. thuringiensis*, *C. glutamicum*) and Gram-negative (*A. tumefaciens*, *E. coli*, *R. leguminosarum*, *B. japonicum*) bacteria strains was studied using the disk diffusion method. Even though this method is not appropriate for the identification of the minimum inhibitory concentration (MIC) of the substance, it allows quick and accurate estimation of the antibacterial activity of preparations on multiple bacterial specimens.^65^

**Fig. 6:**
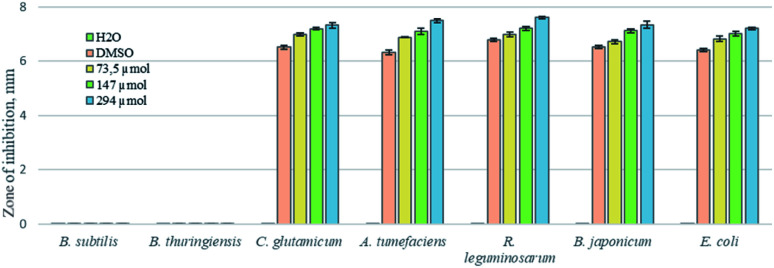
Antibacterial effect of different concentrations of Au NPs on *B. subtilis*, *B. thuringiensis*, *C. glutamicum*, *A. tumefaciens*, *E. coli*, *R. leguminosarum*, *B. japonicum* detected by the disk diffusion assay.

**Fig. 7:**
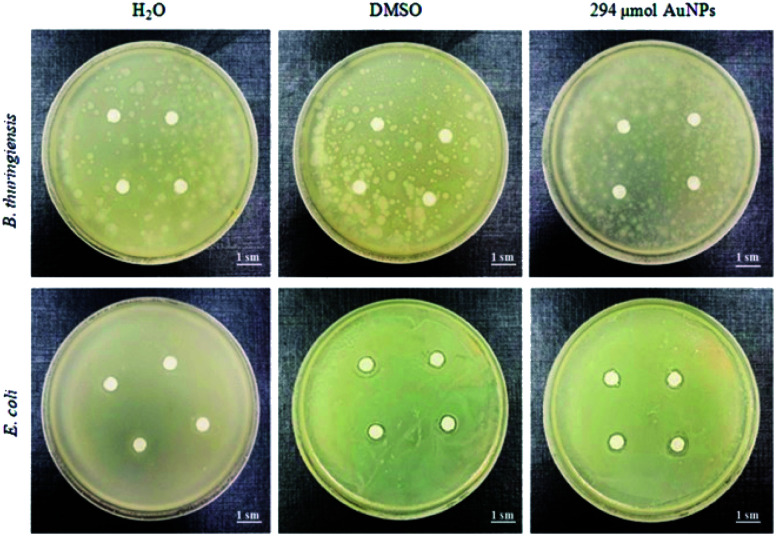
Zones of inhibition assay for antimicrobial activity of DMSO and Au NPs (stock solution) for Gram-positive (*B. thuringiensis*) and Gram-negative (*E. coli*) bacteria.

The results of the disk diffusion assay show that Au NPs do not affect Gram-positive *B. subtilis* and *B. thuringiensis*, and slightly inhibit the growth of *A. tumefaciens* GV3101, *E. coli* DB31, *R. leguminosarum* NRRL B-4403, and *B. japonicum* IMB B-7538 compared with the positive control (DMSO) (Fig. 6 and 7 show Au NPs effects on two species only). Thus, it was found that for the Gram-positive bacterium *C. glutamicum* and all tested Gram-negative species the growth inhibition zones for Au NPs ranged from 6.8 to 7.4 mm in diameter, whereas for DMSO – 6.4–6.7 mm (Fig. 6 and 7).

It is known that the antibacterial properties of Au NPs mainly depend on their concentration, size, shape, surface modifications, presence of impurities (Au^+^ and Au^3+^ ions, reducing reagents or surface stabilizers), and tested microorganisms. The choice of bacteria used in our research was because they are representatives of the ecosystem, and are also used in various research and biotechnological developments: industrial species used for the synthesis of amino acids and enzymes (*B. subtilis*, *C. glutamicum*),^66,67^*B. thuringiensis* producing insecticide and nematicide Bt-proteins, the product of expression of *cry* genes, symbiotic nitrogen fixing bacteria (*R. leguminosarum*, *B. japonicum*),^68^ and model bacteria *A. tumefaciens* and *E. coli* frequently used in biotechnology and molecular biology. Moreover, *B. subtilis* and *B. thuringiensis* are components of soil microbiome,^66^*A. tumefaciens* is a plant pathogenic bacterium, *R. leguminosarum* and *B. japonicum* are nitrogen fixing symbiotic bacteria important for plant growth and soil remediation.^68,69^

In our study, the difference found in sensitivity to Au NPs between Gram-positive and Gram-negative bacteria could be due to the structure of their cell walls and because of their species.^67^ In general, the results obtained are in agreement with the data of other similar research. Most of the studies report the low antibacterial properties (about 10 times lower than that of silver NPs) of naked Au NPs or functionalized with inert chemical compounds such as polyvinyl pyrrolidone (PVP).^70–72^ At the same time, there is a report that shows the antibacterial activity of Au NPs against multiple drug resistant (MDR) bacteria such as bacteria of the «ESKAPE» group (*Escherichia coli*, *Staphylococcus aureus*, *Klebsiella pneumoniae*, *Acinetobacter baumannii*, *Pseudomonas aeruginosa*, *Enterobacter* sp.), which are the most common nosocomial pathogens.^73^ Such Au NPs were produced for possible complex therapy against MRD bacteria, for the detection of markers of different diseases, *etc.*

Thus, here we show for the first time that Au NPs show little or no activity towards bacteria such as *R. leguminosarum*, *B. japonicum*, *B. subtilis*, *C. glutamicum*, *B. thuringiensis*, *A. tumefaciens*. In the previous works, the antimicrobial activity of only Ag NPs or NPs of metal oxides (ZnO, TiO_2_, MgO, *etc.*) on soil microbiome was thoroughly studied in the context of soil contamination.^74,75^

In addition, the solvent is another key factor that determines the antibacterial activity of Au NP solution. Despite the favorable chemical properties of dimethyl sulphoxide (DMSO), this reagent has been rarely used as a solvent or surface stabilizer of metal NPs including Au-based NPs.^76,77^ In our work, DMSO was used also for Au NPs synthesis primarily as a solvent, although it may participate in stabilizing the NPs, according to other works on metal NPs^36,76,77^ and partially supported in this work by XPS, which detected tiny residuals of DMSO in dried samples. Recently it was shown that DMSO influences different cellular processes such as apoptosis, autophagy, cell cycle, differentiation, lipid metabolism, *etc.*^78^ At high concentrations, it enhances cell permeability causing deformation of membranes and disrupting their integrity.^79^ Numerous studies are devoted to the influence of different concentrations of DMSO on the growth of bacteria cultures. For example, in a work by Wadhwani *et al.* (2008)^80^ it was shown that low DMSO concentrations (1–3%) do not affect significantly the growth of 5 different bacterial species (*S. epidermidis*, *P. oleovorans*, *Vibrio cholerae*, *Shigella flexneri*, *S. paratyphi*); the level of bacterial growth was at 93–97% compared to that of the control. Recently, Dyrda *et al.*^81^ showed that the treatment with 20% DMSO resulted in the absolute loss of viability of the cells of *E. coli*, *B. subtilis*, and *Saccharomyces cerevisiae*. In our study, the treatment with 100% DMSO lead to insignificant growth inhibition of *C. glutamicum*, *A. tumefaciens*, *E. coli*, *R. leguminosarum*, *B. japonicum* only, both *Bacillus* species, *B. subtilis* and *B. thuringiensis* were insensitive to DMSO.

Summarizing the data obtained we can conclude that the AuNPs produced by us do not possess such side-effect as pronounced antibacterial activity on the tested non-pathogenic Gram-positive or Gram-negative bacteria as *B. subtilis* and *B. thuringiensis*, *C. glutamicum*, *A. tumefaciens*, *E. coli*, *R. leguminosarum* and *B. japonicum*.

## Conclusions

A new method of wet synthesis of plasmonic Au NPs was developed. This room temperature approach is based on the classical Turkevich synthesis, using sodium citrate as a reducing and stabilizing agent, resulting in highly stable colloidal suspensions of Au NPs in DMSO, with a mean NP size of about 15 nm and fairly low size distribution. The stability test through UV-vis absorption spectroscopy indicates no sign of aggregation for months. Based on the spectral data of XPS and FTIR, the stabilisation mechanism of the Au NP in DMSO is concluded to be similar to that of NPs synthesized in water. The tiny residuals of DMSO in dried samples (detected by XPS) do not hinder the detection of organic analytes by SERS. The obtained Au NPs do not possess such side effect as pronounced antibacterial activity, based on the tests performed on non-pathogenic Gram-positive or Gram-negative bacteria such as *B. subtilis* and *B. thuringiensis*, *C. glutamicum*, *A. tumefaciens*, *E. coli*, *R. leguminosarum* and *B. japonicum*.

## Author contributions

SP, OK, OY – methodology, investigation, data curation; AB – investigation, writing; YP and VD – project administration, funding acquisition, review; AY and VY – conceptualization, editing, critical remarks; DRTZ – writing, review, and editing. All authors have approved the final manuscript version.

## Conflicts of interest

The authors declare that they have no competing interests.

## Supplementary Material
